# Use of flexible intramedullary nailing in combination with an external fixator for a postoperative defect and pseudarthrosis of femur in a girl with osteogenesis imperfecta type VIII: a case report

**DOI:** 10.1007/s11751-018-0320-3

**Published:** 2018-09-29

**Authors:** Dmitry Popkov

**Affiliations:** Russian Ilizarov Scientific Center for Restorative Traumatology and Orthopaedics, 6, M.Ulyanova Street, Kurgan, Russian Federation 640014

**Keywords:** Osteogenesis imperfecta type VIII, Flexible intramedullary nailing, External fixation

## Abstract

Telescopic rodding has been developed in order to obtain long-lasting osteosynthesis in the growing long bones of children with osteogenesis imperfecta (OI). The major and still unsolved drawback of any telescopic rod or nail design is a lack of rotational stability and, currently, no telescopic system allows immediate weight-bearing. When these problems are associated with insufficient longitudinal bone stability and diminished healing capacity, the result can be unfavourable causing secondary bone fragment displacement, delayed or non-union. This article presents a case report of operative treatment in a 9-year-old girl affected with OI type VIII complicated with postoperative defect and pseudarthrosis of the femur causing functional impairment with loss of walking ability. A combination of intramedullary flexible nailing and minimal external fixation was applied for treatment of femoral defect-pseudarthrosis in a girl of 9 years with OI type VIII. Intramedullary and extramedullary nails with wrapping of titanium nickel mesh subperiosteally provided osteosynthesis and deformity correction of the tibia of a small intramedullary canal diameter. Upright standing and walking with progressive weight-bearing was started 4 days after surgery. There were no septic or vascular complications nor complications related to Ilizarov fixator. Radiographs demonstrated bone union in the femur 46 days after surgery. At the follow-up visit 9 months after fixator removal, clinical alignment remained excellent without any relapse of deformity. Bone remodelling with restitution of medullary canal was noted on lower limb radiographs. The patient was able to stand and walk without pain with an aide or walker. At the follow-up visit 17 months after fixator removal, there was no decrease in achieved functional abilities and the treatment outcome remained satisfactory. Use of an external fixator with intramedullary nailing for treatment of postoperative pseudarthrosis in patient with severe OI (recessive form of OI, type VIII) provides longitudinal, rotational and angular stability. Furthermore, this approach ensured early functional activity and walking with full weight-bearing, both favourable conditions for bone tissue regeneration. The external fixator was applied for a short period and only for additional stability and not for progressive deformity correction or other manipulation. In addition, the combination of intramedullary and extramedullary nailing and subperiosteal titanium nickel mesh seems to be promising for osteosynthesis in the deformity correction of bones with small diameter in children with OI.

## Introduction

Osteogenesis imperfecta (OI) is a group of genetic disorders with wide phenotypic and molecular heterogeneity. The major orthopaedic features are bone fragility, osteopenia, progressive bone deformity and varying degrees of short stature [1, 2]. Reported birth prevalence of osteogenesis imperfecta is of 0.3–0.7 per 10,000 [3, 4] or 1 in 10,000–1 in 20,000 births [1, 2, 5].

The classification by Sillence is the most widely used and is based on modes of inheritance, radiological and clinical findings and includes OI types I (mild non-deforming), II (perinatal lethal), III (severe), IV (moderate to severe) [6]. There are 18 known genetically and largely clinically distinct OI types [7, 8]. Recessive types of OI result from mutations in the cartilage-associated protein gene (type VII) and the prolyl 3-hydroxylase 1 gene (type VIII). Types VII and VIII of OI vary from lethal-to-severe bone dysplasia [9–11]. Genes responsible for type VII and VIII of OI represent 5–7% or less than 10% of the disease [11, 12].

Orthopaedic surgery is part of the multidisciplinary approach providing correction of long bone bowing, rotational malalignment, angular deformity and preventing fractures [13–17]. Telescopic rodding and nailing were developed to obtain a long-lasting osteosynthesis in a growing long bone [18, 19]. The major and still unsolved drawback of any telescopic rod or nail design is the lack of rotational stability [16, 20, 21]. Furthermore, all telescopic systems do not facilitate immediate weight-bearing [15, 20, 22] and when these problems are associated with insufficient longitudinal bone stability and diminished healing capacity, the result may be unfavourable from secondary bone fragment displacement, delayed or non-union [15, 20–24].

This article presents a case report of operative treatment of a 9-year-old girl affected with osteogenesis imperfecta type VIII, who underwent a combined surgical technique (FIN associated with Ilizarov fixator) for union of an iatrogenic defect-pseudarthrosis of the femur and simultaneous ipsilateral tibial deformity correction.

## Materials and methods

A girl of 9 years 2 months old (height 101 cm, weight 21.4 kg) presented with walking failure, functional impairment, lower limb length discrepancy, loss of standing ability, bowing of right tibia and a 22-month history of right hip pain related to flexion, extension and rotation movement. Pain and instability of the right leg caused a loss of capacity for upright standing with weight bearing the need for crutches. Pain and functional failure had been progressive over the last 18 months.

The past medical history was significant for multiple fractures managed conservatively. The diagnosis of OI type VIII had been established using targeted next-generation sequencing approach. The family history of the patient is not clear because her parents were unknown, and the child was adopted at the age of 3 years.

Surgery for lower limb deformity correction and intramedullary nailing was undertaken abroad at the age of 7 years. According to medical records, an intramedullary telescopic rod of Fassier-Duval type was inserted into the left femur and tibia. Intramedullary Rush rods were used for right leg (femur and tibia). With medical treatment, the last bisphosphonate dose (pamidronate) was given 6 months prior to admission.

**Fig. 1:**
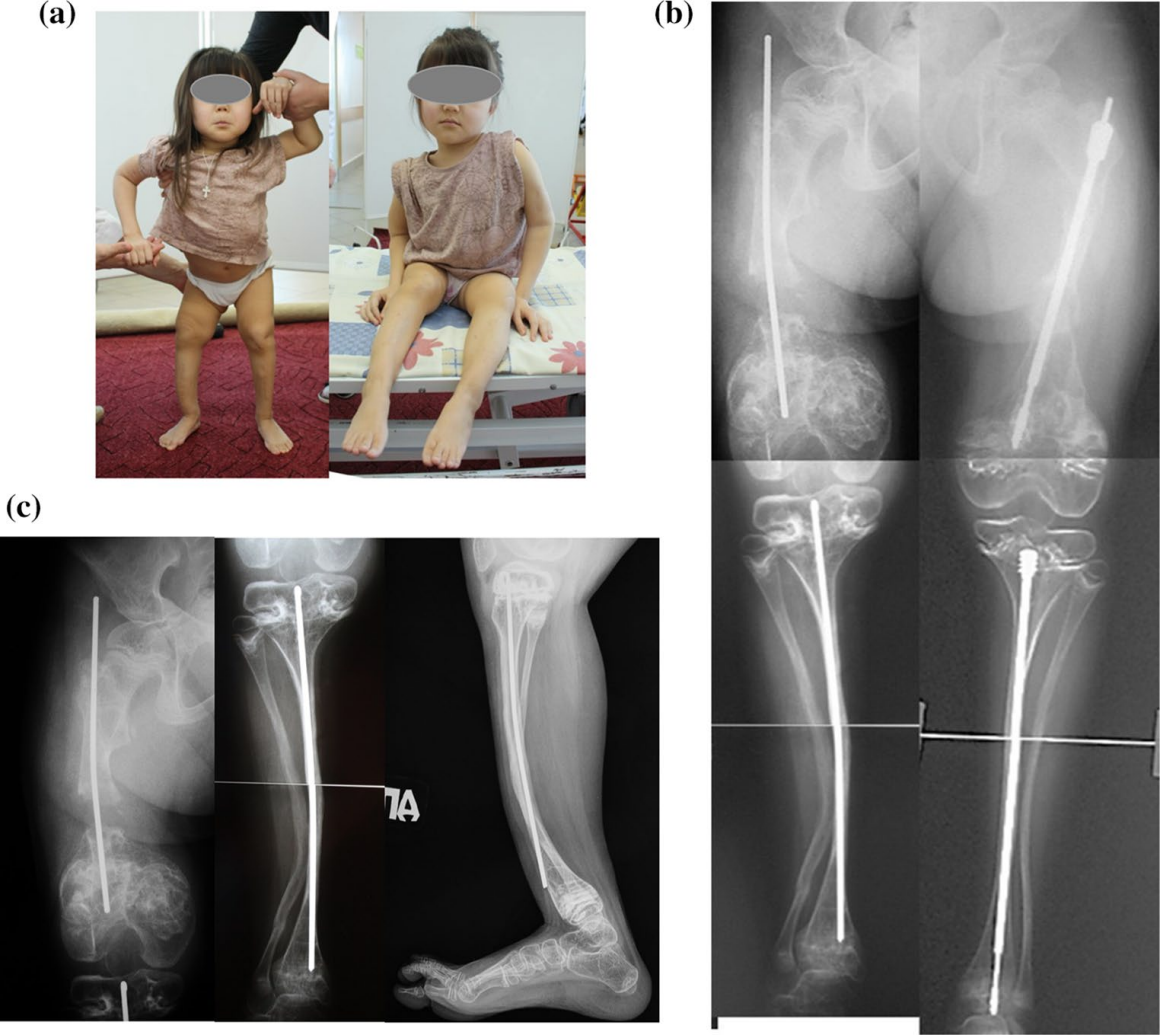
Girl, 9 years old with OI type VIII, before surgery: **a** patient before surgery; **b** standing anteroposterior lower limb radiographs; **c** bone defect and non-union of right femur after excessive bone resection (longitudinal instability of osteosynthesis with Rush rod), proximal migration of Rush rod, valgus-procurvatum deformity of right tibia with external migration of distal extremity of Rush rod through cortex

Clinical examination revealed short stature with rhizomelia (Fig. 1), relative macrocephaly, white sclerae, low degree of myopia, mild hearing loss.

Examination showed that the patient had evident lower limb length discrepancy of 3 cm. Right hip flexion, rotation and abduction were painful with limited ROM: flexion/extension—50°/0/10°; external rotation/internal rotation—30°/0/40°; abduction/adduction—25°/0/40°. Pain was localised over the right hip, proximal femur and knee joint region. Abnormal rotation and angular mobility were found at the distal right femur. The ranges of motion in both knees and ankles were not limited.

The FAQ score (Gillette Functional Assessment Questionnaire Ambulation Scale (FAQ) [25]) was 3 demonstrating that the patient was able to make some steps only with permanent assistance. Laboratory tests were within normal limits, including the C-reactive protein (9.4 mg/L), but there was moderate anaemia (erythrocytes 4.0 × 10^6^ ml; haemoglobin 110 g/L).

X-ray examination revealed diffuse osteoporosis affecting pelvis and long bones with large metaphyses reflecting growth plate disorganization (Fig. 1). There were zones of popcorn calcification and areas of disorganized hyperdense lines in the metaphysis and epiphysis around the growth plate. Fassier-Duval telescopic rods were identified in the left femur and tibia, but there were signs of the implants were not telescoping and these were associated with large areas of epiphysiodesis in distal femur and proximal tibia.

On the right side, we noted proximal migration of the intramedullary Rush nail explaining mechanical pain and limited ROM in the right hip. Furthermore, there was a defect-pseudarthrosis with gap of 22 mm at distal femur. We hypothesized that excessive bone resection and longitudinal and rotational instability resulted in non-union and diastasis between the fragments. Valgus (9°) and procurvatum (32°) deformity of right tibia was observed causing anterior migration of the distal part of tibial Rush nail through anterior cortex. Radiographic evaluations of angles were made preoperatively on the supine position: mLDFA—91° (R), 94° (L); mMPTA—95°(R), 90° (L); mLDTA—84°(R), 85° (L).

### Surgical technique

**Fig. 2:**
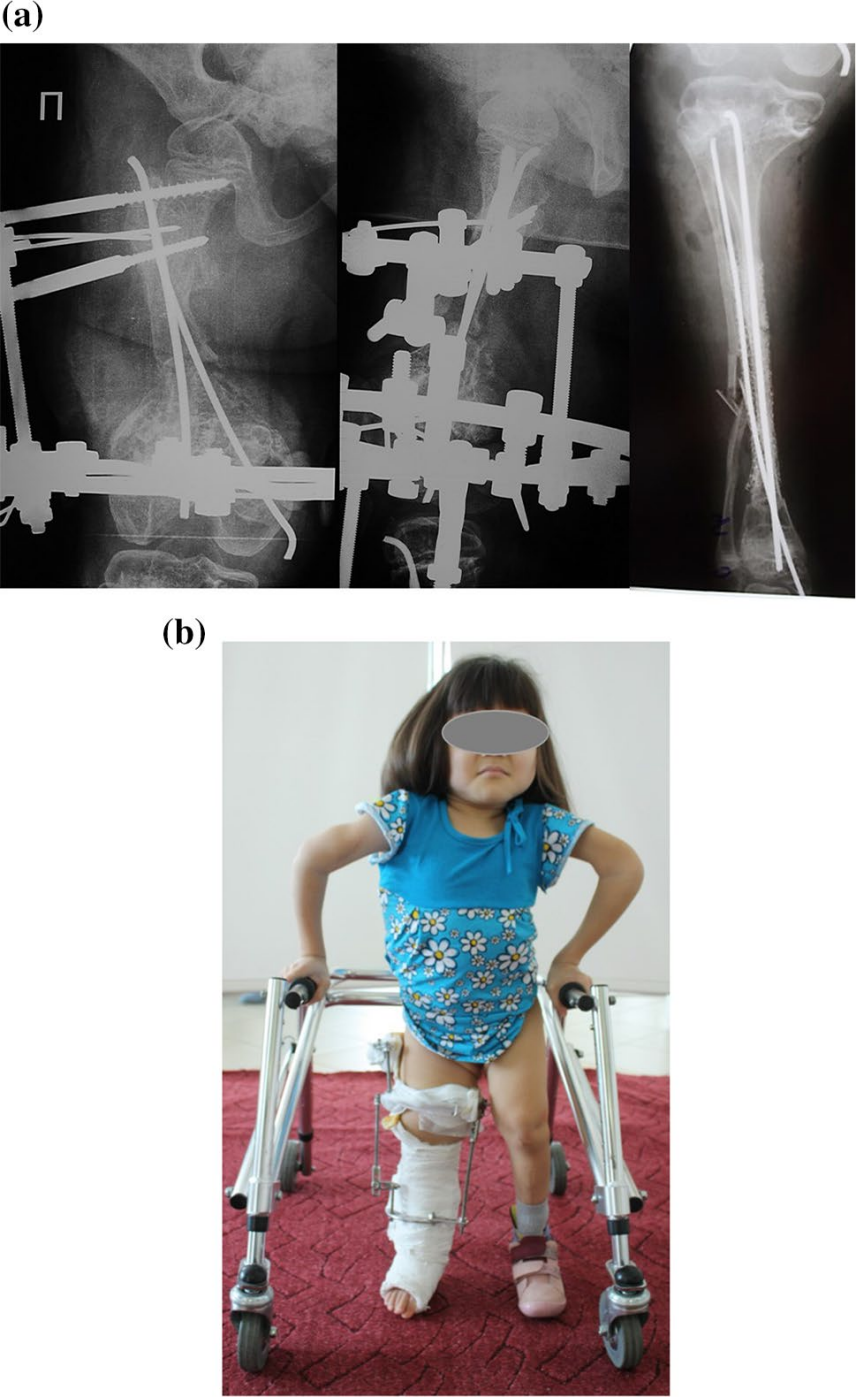
Surgery and postoperative period: **a** intraoperative radiographs of right femur and tibia; surgery consisted of Rush rod removal; FIN of femur associated with Ilizarov fixator and FIN of tibia associated with subperiosteal titanium nickelide mesh; plaster cast was applied at tibia and foot and integrated with Ilizarov fixator; **b** patient standing with walker in early postoperative period

For the correction of problems on right femur and tibia, a simultaneous approach to both segments was undertaken (Fig. 2). The surgery in the femur consisted of Rush rod removal, open limited resection of the pseudarthrosis ends (open technique using oscillating power saw) and bipolar flexible intramedullary nailing (FIN) of the femur and application of the Ilizarov fixator. The external fixator assembly included a short proximal arc (proximal metaphysis level) with two half-pins and two half-wires and a distal ring (distal metaphysis level) where three wires with olives were placed and tensioned. Two titanium flexible nails of 3 mm and 2.5 mm diameters were inserted through proximal and distal short incisions. In the tibia, removing of the Rush nail was followed by closing wedge osteotomy of tibia and bipolar titanium flexible intramedullary nailing. A fibula osteoclasis was performed by applying direct force to the bone before completion of the tibial osteotomy. An antegrade tibial nail of 3 mm diameter was inserted. A retrograde nail was then inserted through the medial malleolus and placed subperiosteally at the level of middle and proximal parts of diaphysis due to small diameter of the medullary canal. Then, its leading end was inserted into bone under direct vision at the level of the opposite proximal metaphysis. The curvatures of nails were re-oriented in the opposite directions to the residual angular deformity. This tibial nailing was complemented by a subperiosteal application of titanium nickel mesh (TN-10; 150 μm) wrapping the bone fragments and nails together. On the 3rd postoperative day, a plaster cast was applied on the tibia and foot and integrated to the Ilizarov fixator using threaded rods.

Upright standing and walking with progressive weight-bearing was started 4 days after surgery. There were no septic or vascular complications. The patient was discharged from hospital 2 weeks after surgery. She was advised to maintain an adequate calcium intake and physical activity with emphasis on walking full weight-bearing.

## Results

**Fig. 3:**
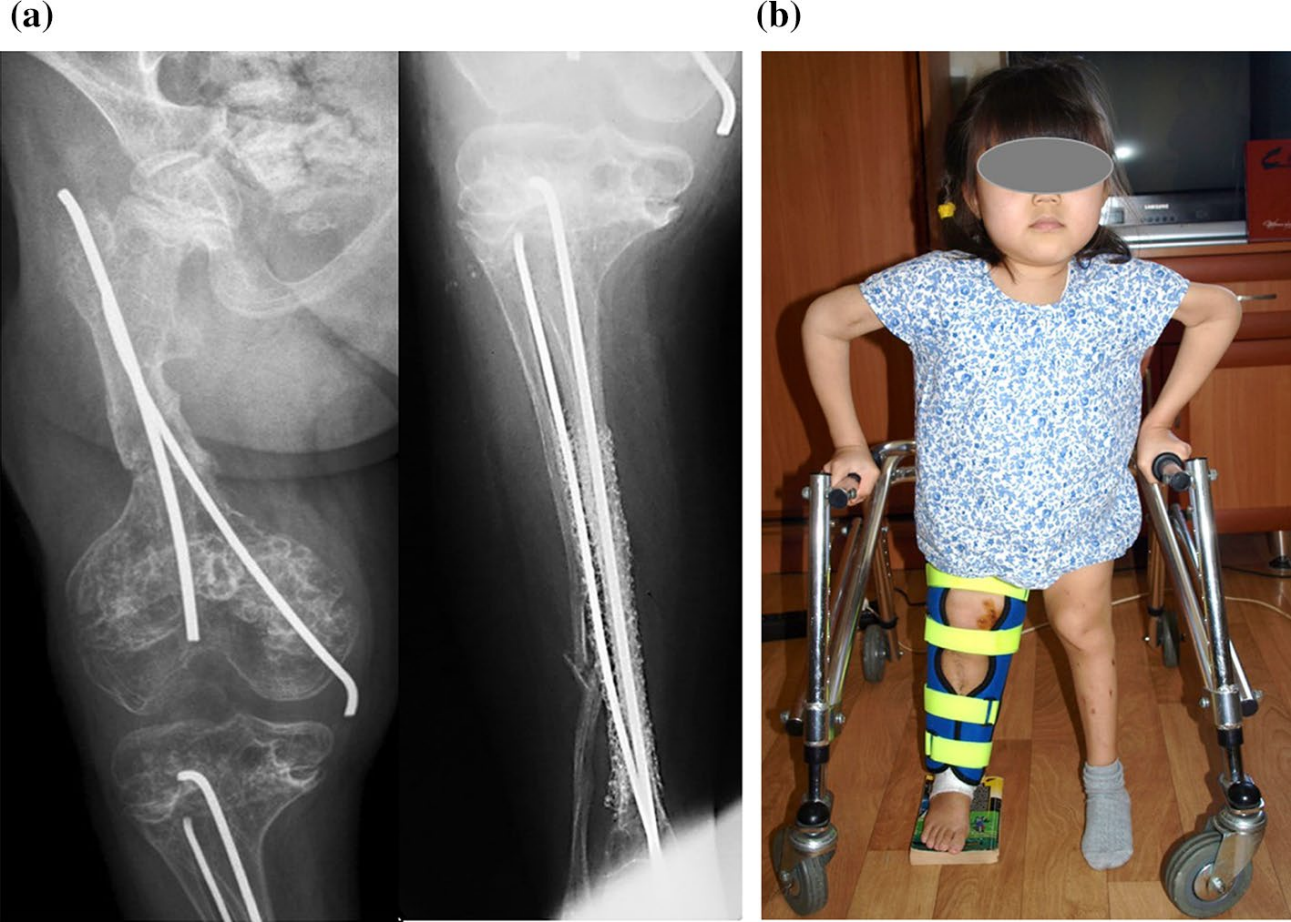
After fixator removal: **a** X-radiographs of right femur and tibia on the day of external fixator removal; **b** patient in 2 days after fixator removal

Radiographs demonstrated bone union in femur 46 days after surgery. The fixator was removed under GA (Fig. 3). Radiographic evaluations of angles at right side demonstrated an mLDFA—93°; mMPTA—87°. An orthotic was used after fixator removal and maintained for 3 weeks.

**Fig. 4:**
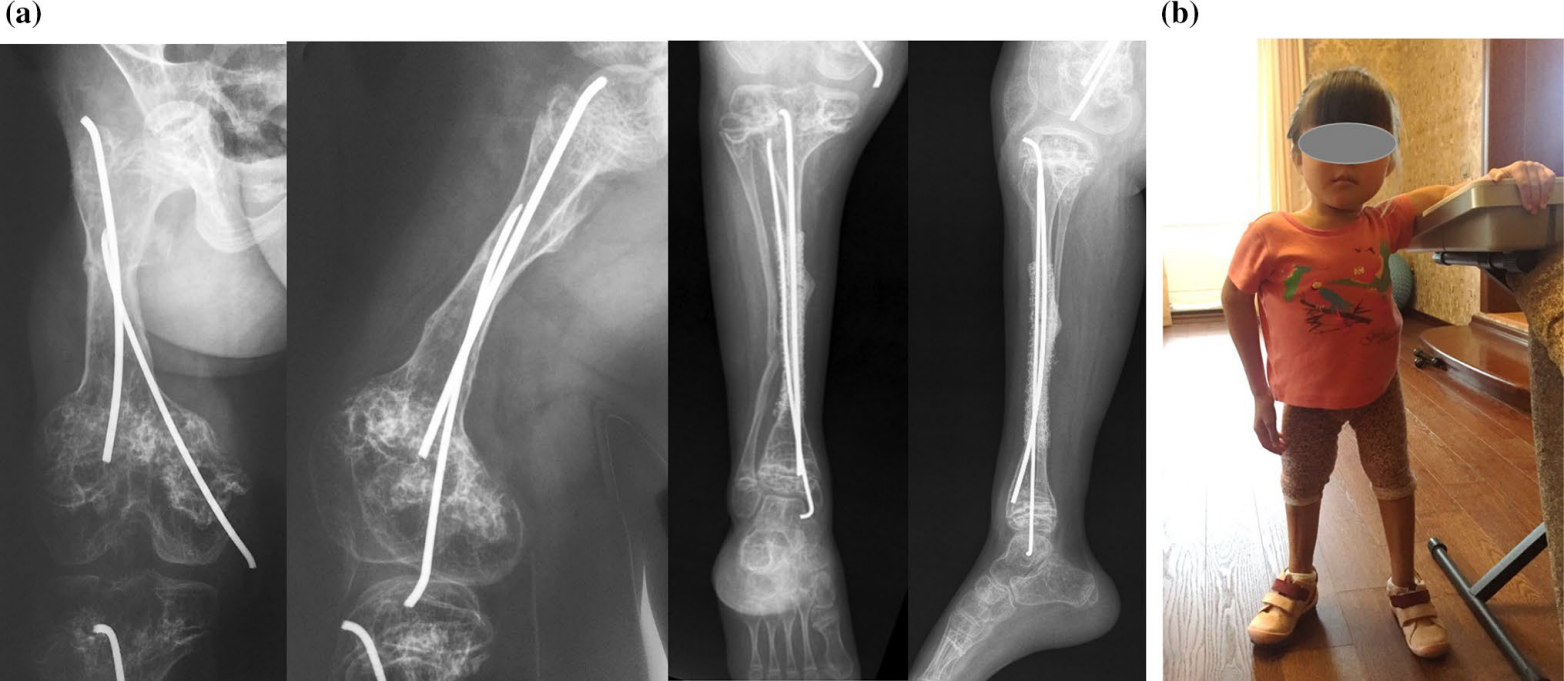
In 9 months after fixator removal: **a** radiographs of right femur and tibia, note remodelling of callus at femur and large periosteal callus at tibia covering titanium mesh; **b** patient standing with full weight-bearing

At the follow-up visit 9 months (Fig. 4) after fixator removal, clinical alignment remained excellent without any recurrence of deformity. Bone remodelling with restitution of the medullary canal was noted on the radiographs. Radiographic evaluations of angles on the right side were: mLDFA—94°; mMPTA—92°; mLDTA—84°. The values were close to the ones measured after fixator removal. Telescoping of the intramedullary nails indicated longitudinal growing of the femur.

The patient was able to stand and work with an aide or walker and without pain. The range of motion in the hip and knee joints was not restricted. Bisphosphonate treatment had been resumed at that moment.

At the follow-up visit 17 months after fixator removal, there was no decrease in functional abilities. At 1.5 years postoperatively, the FAQ score was 6.

## Discussion

Osteogenesis imperfecta is a rare genetic connective tissue disorder with a dominant or recessive inheritance pattern and with the primary clinical manifestation involving the skeleton characterized by osteoporosis and increased susceptibility to fracture [6, 8, 11, 12]. Type VIII of OI is recessive and was identified in 2006 [9, 26, 27]. Null mutation in prolyl 3-hydroxylase 1 causes type VIII of OI with severe-to-lethal bone dysplasia and over modification of the type I collagen helical region [8, 11]. Recessive types cause OI in less than 10% of cases [11, 12]. Recessive types VII and VIII are undistinguishable in children. Both have rhizomelia, undertubulation of long bones, white sclerae, changed head circumference, short stature and progressive deformities [8, 11]. A case of bilateral giant retinal tears was reported in a 9-year-old boy with type VIII of OI [7]. Obafemi AA et al. [28] reported “popcorn” calcification around the growth plate on lower limb radiographs (distal femur and proximal tibia) of two children with type VIII of OI. In patients with confirmed type III OI, this radiological feature was also found in 52% of cases. Authors have emphasized that the popcorn calcification is likely to be frequent in severe OI but do not distinguish cases with defects in collagen structure (dominant type III of OI) or related to collagen synthesis (recessive type VIII). Our reported case clinically and radiologically corresponds to a severe but not lethal type of OI with only targeted next-generation sequencing identifying this type VIII of OI.

A better quality of life including improved mobility and functional independence are the main goals of treatment for children with OI [1, 29–31]. These therapeutic plans should be based on a long-term multidisciplinary approach, which includes medical treatment with bisphosphonates, orthopaedic treatment for fractures and deformity correction and rehabilitation [14, 29, 30, 32, 33].

The benefits of a telescoping intramedullary rod in fractures and deformity of long bones in children with OI are well known [16, 17, 19, 34]. The major and unsolved inconvenience of any telescopic system design is the lack of rotational stability [16, 20, 21]. In children with OI, this problem can be associated with insufficient longitudinal bone stability (in the case of shortening of a segment at surgery or due to weakness of bone tissue) [21, 24] and diminished healing capacity [22]. Furthermore, all telescoping systems are not implants for immediate weight-bearing. Only when radiological evidence of callus formation is present can active physiotherapy and weight-bearing with the aid of orthoses be started [15, 20, 22]. In children with severe forms of OI, the long bones are narrow and often not suitable for telescoping rods [13, 35]. In such a situation, regular or elastic nailing achieves the required outcomes [16, 21, 24, 36].

The reported reoperation rate in severe and moderate-to-severe forms of OI treated with telescoping rods varies from 13 to 53% depending on the average follow-up and the segment operated [15, 20, 23, 37, 38]. The use of sliding FIN in children with OI increased the reoperation rate to 75% at 8 years’ follow-up [24]. In regular rod application (non-sliding), the reoperation rate is 58–87% [39, 40].

Complications of with all types of rods are not rare. These include non-telescoping failures, joint intrusion, migration of parts of rod, fractures, delayed or non-union, bent nails and iatrogenic epiphysiodesis; overall a 35–40%-complication rate was reported [15, 20, 24, 39, 41]. Mechanical complications remain the main problem in all published series [15, 20, 24]. The rate of non-union and delayed union varies from 0 to 14.5% [20, 23]. Boutard and Laville report one case of pseudarthrosis (femur) from a series of 14 patients [24]. Munns et al. observed delayed bone healing in 103 out of 200 interventions [22]. And this complication was more frequent in patients receiving pamidronate, in children with OI type IV and from osteotomy of the tibia.

Using an external fixator as an additional stabilization element in the treatment of orthopaedic problems from metabolic bone disorders is not new [20, 42–44]. Birke et al. [20] reported use of the Ilizarov fixator to be beneficial as additional stabilization to telescoping rodding in patients with severe underlying bone pathology but did not describe experience with the combined technique in OI. Kong and Sabharwal used FIN and monolateral external fixation in the treatment of femoral shaft fractures in children with OI; the external fixator provided angular and torsional stability at the fracture site and avoided inconvenient supplemental casting [44].

In this case, the combination of FIN and Ilizarov fixator ensured stability for the femoral bone fragments and permitted early full weight-bearing in the immediate postoperative period. That resulted in bone union at the pseudarthrosis site within 45 days. There was no secondary torsional deformity in postoperative period. This approach represents an advantage in comparison with the use of telescopic rodding alone. With regard to the tibial deformity correction, a combination of intramedullary flexible nail, subperiosteal flexible nail and subperiosteal titanium net seems to be useful in small diameter shaft bones. The achievement of all normal radiological parameters of anteroposterior alignment of lower limbs [45] in children with severe forms of OI may be improbable, but overall realignment should be considered as crucial for the goal of better quality of life including mobility and functional independence.

## Conclusion

We found that the use of an Ilizarov fixator combined with FIN in treating a postoperative pseudarthrosis of the femur in a patient with severe OI (recessive form of OI, type VIII) provided longitudinal, rotational and angular stability. This approach ensured early functional activity and full weight-bearing, both favourable conditions for bone tissue regeneration. We should emphasize that the external fixator was applied for a short period only and for additional stability and not for progressive deformity correction or other manipulation. The combination of intramedullary and subperiosteal nailing together with subperiosteal titanium nickel mesh also seems to be promising for osteosynthesis in deformity correction of small diameter bones in children with OI.

## References

[1] Bregou Bourgeois A, Aubry-Rozier B, Bonafé L, Laurent-Applegate L, Pioletti DP, Zambelli PY (2016). Osteogenesis imperfecta: from diagnosis and multidisciplinary treatment to future perspectives. Swiss Med Wkly.

[2] Folkestad L, Hald JD, Ersbøll AK, Gram J, Hermann AP, Langdahl B, Abrahamsen B, Brixen K (2016). Fracture rates and fracture sites in patients with osteogenesis imperfecta—a nationwide register-based cohort study. J Bone Miner Res.

[3] Orioli IM, Castilla EE, Barbosa-Neto JG (1986). The birth prevalence rates for the skeletal dysplasias. J Med Genet.

[4] Stevenson DA, Carey JC, Byrne JL, Srisukhumbowornchai S, Feldkamp ML (2012). Analysis of skeletal dysplasias in the Utah population. Am J Med Genet A.

[5] Van Dijk FS, Sillence DO (2014). Osteogenesis imperfecta: clinical diagnosis, nomenclature and severity assessment. Am J Med Genet A.

[6] Sillence DO, Senn A, Danks D (1979). Genetic heterogeneity in osteogenesis imperfecta. J Med Genet.

[7] Scollo P, Snead MP, Richards AJ, Pollitt R, DeVile C (2018). Bilateral giant retinal tears in osteogenesis imperfecta. BMC Med Genet.

[8] Marini JC, Forlino A, Bächinger HP, Bishop NJ, Byers PH, Paepe A, Fassier F, Fratzl-Zelman N, Kozloff KM, Krakow D, Montpetit K, Semler O (2017). Osteogenesis imperfecta. Nat Rev Dis Primers.

[9] Cabral WA, Chang W, Barnes AM, Weis M, Scott MA, Leikin S, Makareeva E, Kuznetsova NV, Rosenbaum KN, Tifft CJ, Bulas DI, Kozma C, Smith PA, Eyre DR, Marini JC (2007). Prolyl 3-hydroxylase 1 deficiency causes a recessive metabolic bone disorder resembling lethal/severe osteogenesis imperfecta. Nat Genet.

[10] Pepin MG, Schwarze U, Singh V, Romana M, Jones-Lecointe A, Byers PH (2013). Allelic background of LEPRE1 mutations that cause recessive forms of osteogenesis imperfecta in different populations. Mol Genet Genom Med.

[11] Chang W, Barnes AM, Cabral WA, Bodurtha JN, Marini JC (2010). Prolyl 3-hydroxylase 1 and CRTAP are mutually stabilizing in the endoplasmic reticulum collagen prolyl 3-hydroxylation complex. Hum Mol Genet.

[12] Árvai K, Horváth P, Balla B, Tobiás B, Kató K, Kirschner G, Klujber V, Lakatos P, Kósa JP (2016). Next-generation sequencing of common osteogenesis imperfecta-related genes in clinical practice. Sci Rep.

[13] Enright W, Noonan K (2006). Bone plating in patients with type III osteogenesis imperfecta: results and complications. Iowa Orthop J.

[14] Roberts TT, Cepela DJ, Uhl RL, Lozman J (2016). Orthopaedic considerations for the adult with osteogenesis imperfecta. J Am Acad Orthop Surg.

[15] Ruck J, Dahan-Oliel N, Montpetit K, Rauch F, Fassier F (2011). Fassier-Duval femoral rodding in children with osteogenesis imperfecta receiving bisphosphonates: functional outcomes at one year. J Child Orthop.

[16] Sterian A, Balanescu R, Barbilian A, Tevanov I, Carp M, Nahoi C, Barbu M, Ulici A (2015). Early telescopic rod osteosynthesis for osteogenesis imperfecta patients. J Med Life.

[17] Zeitlin L, Fassier F, Glorieux FH (2003). Modern approach to children with osteogenesis imperfecta. J Pediatr Orthop B.

[18] Georgescu I, Vlad C, Gavriliu TŞ, Dan S, Pârvan AA (2013). Surgical treatment in osteogenesis imperfecta—10 years experience. J Med Life.

[19] Violas P, Mary P (2008). Imperfecta osteogenesis: interest of surgical treatment. Arch Pediatr.

[20] Birke O, Davies N, Latimer M, Little DG, Bellemore M (2011). Experience with the Fassier-Duval telescopic rod: first 24 consecutive cases with a minimum of 1-year follow-up. J Pediatr Orthop.

[21] Sterian A, Balanescu R, Barbilian A, Ulici A (2015). Osteosynthesis in osteogenesis imperfecta, telescopic versus non-telescopic nailing. J Med Life.

[22] Munns CF, Rauch F, Zeitlin L, Fassier F, Glorieux FH (2004). Delayed osteotomy but not fracture healing in pediatric osteogenesis imperfecta patients receiving pamidronate. J Bone Miner Res.

[23] Azzam KA, Rush ET, Burke BR, Nabower AM, Esposito PW (2016). Mid-term results of femoral and tibial osteotomies and Fassier-Duval nailing in children with osteogenesis imperfecta. J Pediatr Orthop.

[24] Boutaud B, Laville JM (2004). Elastic sliding central medullary nailing with osteogenesis imperfecta. Fourteen cases at eight years follow-up. Rev Chir Orthop Reparatrice Appar Mot.

[25] Novacheck TF, Stout JL, Tervo R (2000). Reliability and validity of the Gillette Functional Assessment Questionnaire as an outcome measure in children with walking disabilities. J Pediatr Orthop.

[26] Barnes AM, Chang W, Morello R, Cabral WA, Weis M, Eyre DR, Leikin S, Makareeva E, Kuznetsova N, Uveges TE, Ashok A, Flor AW, Mulvihill JJ, Wilson PL, Sundaram UT, Lee B, Marini JC (2006). Deficiency of cartilage-associated protein in recessive lethal osteogenesis imperfecta. N Engl J Med.

[27] van Dijk FS, Nesbitt IM, Zwikstra EH, Nikkels PG, Piersma SR, Fratantoni SA, Jimenez CR, Huizer M, Morsman AC, Cobben JM, van Roij MH, Elting MW, Verbeke JI, Wijnaendts LC, Shaw NJ, Högler W, McKeown C, Sistermans EA, Dalton A, Meijers-Heijboer H, Pals G (2009). PPIB mutations cause severe osteogenesis imperfecta. Am J Hum Genet.

[28] Obafemi AA, Bulas DI, Troendle J, Marini JC (2008). Popcorn calcification in osteogenesis imperfecta: incidence, progression, and molecular correlation. Am J Med Genet A.

[29] Dahan-Oliel N, Oliel S, Tsimicalis A, Montpetit K, Rauch F, Dogba MJ (2016). Quality of life in osteogenesis imperfecta: a mixed-methods systematic review. Am J Med Genet A.

[30] Dogba MJ, Rauch F, Wong T, Ruck J, Glorieux FH, Bedos C (2014). From pediatric to adult care: strategic evaluation of a transition program for patients with osteogenesis imperfecta. BMC Health Serv Res.

[31] Montpetit K, Palomo T, Glorieux FH, Fassier F, Rauch F (2015). Multidisciplinary treatment of severe osteogenesis imperfecta: functional outcomes at skeletal maturity. Arch Phys Med Rehabil.

[32] Otaify GA, Aglan MS, Ibrahim MM, Elnashar M, El Banna RA, Temtamy SA (2016). Zoledronic acid in children with osteogenesis imperfecta and Bruck syndrome: a 2-year prospective observational study. Osteoporos Int.

[33] Marginean O, Tamasanu RC, Mang N, Mozos I, Brad GF (2017). Therapy with pamidronate in children with osteogenesis imperfecta. Drug Des Dev Ther.

[34] Metaizeau JP (1987). Sliding centro-medullary nailing. Application to the treatment of severe forms of osteogenesis imperfecta. Chir Pediatr.

[35] Sinikumpu JJ, Ojaniemi M, Lehenkari P, Serlo W (2015). Severe osteogenesis imperfecta Type-III and its challenging treatment in newborn and preschool children. A systematic review. Injury.

[36] Abulsaad M, Abdelrahman A (2009). Modified Sofield–Millar operation: less invasive surgery of lower limbs in osteogenesis imperfecta. Int Orthop.

[37] Fassier F, Esposito P, Sponseller P et al (2006) Multicenter radiological assessment of the Fassier–Duval femoral rodding. In: Proceedings of the annual meeting of the pediatric orthopaedic society of North America (POSNA), San Diego, California

[38] Halloran J, Fassier F, Alam N (2010) Radiological assessment of Fassier-Duval tibial rodding in patients with osteogenesis imperfecta. In: Proceedings of the 29th annual meeting of the european paediatric orthopaedic society (EPOS), Zagreb, Croatia

[39] Li WC, Kao HK, Yang WE, Chang CJ, Chang CH (2015). Femoral non-elongating rodding in osteogenesis imperfecta—the importance of purchasing epiphyseal plate. Biomed J.

[40] Joseph B, Rebello G, Chandra Kant B (2005). The choice of intramedullary devices for the femur and the tibia in osteogenesis imperfecta. J Pediatr Orthop.

[41] Popkov DA, Kononovich NA, Mingazov ER, Shutov RB, Barbier D (2015). Intramedullary elastic transphyseal tibial osteosynthesis and its effect on segmental growth. Vestn Ross Akad Med Nauk.

[42] Popkov A, Aranovich A, Popkov D (2015). Results of deformity correction in children with X-linked hereditary hypophosphatemic rickets by external fixation or combined technique. Int Orthop.

[43] Saldanha KA, Saleh M, Bell MJ, Fernandes JA (2004). Limb lengthening and correction of deformity in the lower limbs of children with osteogenesis imperfecta. J Bone Joint Surg.

[44] Kong H, Sabharwal S (2016). Fixator-augmented flexible intramedullary nailing for osteopenic femoral shaft fractures in children. J Pediatr Orthop B.

[45] Popkov D, Lascombes P, Berte N, Hetzel L, Baptista BR, Popkov A, Journeau P (2015). The normal radiological anteroposterior alignment of the lower limb in children. Skeletal Radiol.

